# Analysis of long-term care for older adults in Brazil, Spain, and Portugal: Perspectives and challenges

**DOI:** 10.1016/j.aprim.2026.103466

**Published:** 2026-02-20

**Authors:** Letycia Parreira de Oliveira, Beatriz Aparecida Ozello Gutierrez, Rosa Yuka Sato Chubaci, Maria Liz Cunha de Oliveira, José Manuel Peixoto Caldas, Henrique Salmazo-Silva

**Affiliations:** a Catholic University of Brasília, Stricto Sensu Graduate Program in Gerontology, Brasília, Federal District, Brazil; b University of São Paulo, School of Arts, Sciences and Humanities, Graduate Program in Gerontology, São Paulo, SP, Brazil; c University of Porto, Graduate Program in Public Health, Porto, Portugal; d University of Salamanca, Salamanca, Spain; e Federal University of Paraiba, João Pessoa, PB, Brazil

**Keywords:** Older adults, Health policies, Care, Aging, Personas mayores, Políticas de salud, Cuidados, Envejecimiento

## Abstract

This study analyzed long-term care policies within the universal health systems of Brazil, Spain, and Portugal using a comparative approach that combines structural similarity and policy contrast across different stages of demographic aging and long-term care development, through a documentary and bibliographic review based on legislation, official records, and international health and social indicators from the OECD published between 2019 and 2022. A qualitative comparative analysis of documents and official reports was conducted to identify similarities, differences, and gaps in care policies. The results show that Spain has a more organized structure than Portugal, offering extensive home care services, day centers, and financial support for caregivers, although population aging poses a challenge to system sustainability. In contrast, Brazil still lacks comprehensive policies aimed at older adults with functional limitations. It is concluded that Brazil's long-term care policies need to be redesigned, with an emphasis on strengthening service organization and improving human resource training to meet the growing demands of an aging population.

.

## Abstract (es)

Este estudio analizó las políticas de cuidados de larga duración en el marco de los sistemas universales de salud de Brasil, España y Portugal, utilizando un enfoque comparativo que combina similitud estructural y contraste de políticas en diferentes etapas del envejecimiento demográfico y del desarrollo de los cuidados de larga duración, mediante una revisión documental y bibliográfica basada en legislación, registros oficiales e indicadores internacionales de salud y sociales de la OCDE publicados entre 2019 y 2022. Se realizó un análisis comparativo cualitativo de documentos e informes oficiales para identificar similitudes, diferencias y vacíos en las políticas de cuidados. Los resultados muestran que España presenta una estructura más organizada que Portugal, con amplia oferta de atención domiciliaria, centros de día y apoyo financiero a cuidadores, aunque el envejecimiento poblacional plantea un desafío para la sostenibilidad del sistema. En contraste, Brasil aún carece de políticas integrales dirigidas a personas mayores con limitaciones funcionales. Se concluye que es necesario rediseñar las políticas brasileñas de cuidados de larga duración, fortaleciendo la organización de los servicios y la capacitación de recursos humanos.

## Introduction

Aging is one of the main contemporary demographic transformations, with direct effects on health systems, social protection, and the organization of care.^1^ Projections indicate that the population aged 65 years and over will grow by 41%, increasing from 92.1 million in 2020 to 130.2 million in 2050. During the same period, the population aged 80 years and over is expected to grow by 88%, rising from 26.6 million to 49.9 million.^2^

This scenario intensifies discussions on how the Families, the State, and Society will organize themselves to provide care strategies and Long-Term Care (LTC).^3^, ^4^

LTC is characterized by a variety of medical, social, and healthcare services that focus on the well-being of older people and the maximizing their functional capacity and health. These services are aimed at healthy, independent older adults and/or those with some degree of dependency.^5^ The assessment of domains such as physical and mental health, cognition, psychological well-being, and aspects related to functionality, social support, and the environment^6^ is a fundamental pillar for the planning of actions in the field of aging.

Although population aging affects older adults broadly, long-term care policies are primarily directed at individuals with functional dependency who require continuous support, whether in institutional settings or through formal care services.

Thus, it is necessary to assess social risk and implement effective public policies and formal care outside the family context, such as those offered by Long-Term Care Institutions for Older People (ILPI).^7^

Within this context, the present study investigates the configurations of long-term care policies in three universal healthcare models: Brazil, Spain, and Portugal. It examines the formulation and improvement of national public policies, as well as consolidated experiences from countries at more advanced stages of the demographic transition, such as Portugal and Spain.^8^ The choice of these countries followed a comparative strategy based on both similarity and contrast. All three operate within universal health systems, enabling the analysis of long-term care policies under comparable institutional principles. Spain and Portugal were intentionally selected due to their shared cultural traditions of family-based care and advanced population aging, while presenting relevant differences in the organization, scope, and institutionalization of long-term care policies. Brazil was included as a contrasting case, representing a country with a universal health system but an emerging and still fragmented long-term care framework. This approach allows the identification of policy gaps, challenges, and transferable experiences across different stages of long-term care policy development.

Therefore, this study aims to analyze and compare long-term care policies directed at older adults with functional dependency, particularly those aged 80 years and over and/or those requiring continuous care, within the universal health systems of Brazil, Spain, and Portugal, with a focus on institutional and other formal long-term care arrangements.

## Methodology

A documentary and bibliographic research was carried out, based on records and legislation issued by official bodies in Brazil, Spain, and Portugal. The countries were selected based on a comparative strategy combining structural similarity and policy contrast, considering the presence of universal health systems and different stages of long-term care policy development. Primary and secondary sources related to public policies for long-term care targeting older adults with functional dependency were used as reference materials. These policies primarily address individuals aged 80 years and over and/or those requiring continuous care, including both institutionalized older adults and those receiving formal long-term care services in the community. For this purpose, a qualitative documentary analysis was conducted.^9^

Additionally, an analysis was conducted of OECD health and long-term care indicators for the years 2019–2021, was conducted to examine how long-term care policies are associated with demographic aging and care realities in each country. The indicators included: life expectancy; mortality from preventable and treatable causes; mortality from specific causes; number of professionals working in the context of long-term care; and number and type of Long-Term Care Institutions for older people.

The documentary and bibliographic review included both scientific literature and gray literature. Scientific articles were identified through searches in academic databases and journals relevant to public health, aging, and social policy. Gray literature consisted of official legislation, policy documents, governmental reports, and institutional publications related to long-term care in Brazil, Spain, and Portugal.

The selection of documents followed predefined inclusion criteria: (i) relevance to long-term care policies targeting older adults with functional dependency; (ii) official or peer-reviewed sources; and (iii) publication between 2019 and 2022. Documents were excluded when they were not directly related to long-term care, focused exclusively on acute care, or did not address the policy, organization, or regulation of long-term care services. The final corpus of documents was analyzed using a qualitative documentary analysis approach.

The comparative analysis was conducted using a qualitative analytical framework. After document selection, information was organized and compared across countries according to predefined analytical dimensions, including: (i) organization and governance of long-term care policies; (ii) target population and eligibility criteria; (iii) types of formal long-term care services and institutional arrangements; and (iv) workforce availability and institutional coverage. These dimensions allowed for a systematic cross-country comparison, identifying similarities, differences, policy gaps, and stages of long-term care policy development.

## Results and discussion

In addition to differences in demographic aging and long-term care policy development, the three countries analyzed present distinct social and cultural contexts that influence care arrangements. Brazil is characterized by greater socioeconomic inequalities, higher levels of informal employment, and a strong reliance on family-based care, often provided by women, which limits the expansion and formalization of long-term care services.^10^, ^11^ In contrast, Spain and Portugal, despite also maintaining strong family care traditions, have more consolidated welfare systems and greater State involvement in long-term care provision, reflecting historical trajectories of social protection and demographic transition.^8^, ^34^

In the Brazilian context, LTC modalities are mostly concentrated in family care and in Long-Term Care Institutions for Older People (ILPI.^10^, ^11^ There is a significant shortage in the provision of formal care.^12^ In this context, during the Interministerial Conference on LTC held in 2023, the challenges and opportunities for creating a unified national policy were discussed, with the aim of structuring guidelines and legislation to integrate social and health care in Brazil.^13^

One of the main proposals was the need to strengthen, expand, and regulate the long-term care service network, both in the home setting and in Long-Term Care Institutions (ILPI).^14^

Some local experiences demonstrate the potential of these proposals when effectively implemented. In certain Brazilian metropolitan regions, there are already initiatives focused on comprehensive care for older adults. For example, in São Paulo – SP, the Elderly Care Program (PAI) stands out, combining home care, day center services, and social support. In Belo Horizonte – MG, the “Maior Cuidado” Program was implemented, offering home care that includes health services, social support, and family guidance, as well as caregiver training and research to inform the development of more effective public policies.^15^

However, despite these advances, Brazil still faces challenges such as regional inequality, lack of financial resources, and the need for greater coordination among different levels of government.^16^

On the other hand, Portugal has a more developed long-term care system compared to Brazil, standing out for the implementation of the Social Services and Equipment Network (RSES) and the National Network for Integrated Continuous Care (RNCCI)^17^ (Table 1, Table 2). Although family care for older people remains active, the formal network – composed of public–private partnerships – offers day centers that provide care and social activities, in addition to home support and assistance. With a consolidated regulatory and institutional structure, residential facilities for older adults offer accommodation, infrastructure, and long-term care for elderly individuals in situations of dependency.^18^

Although long-term care policies are mainly coordinated through social assistance structures, they are embedded within universal national health systems in all three countries, which provide clinical support, regulation, and integration with social care services (Table 1, Table 2).

**Table 1:** Relationship between Social Services and Equipment (RSES) and National Network for Integrated Continuous Care (RNCCI).

Characteristic	RSES	RNCCI
Framework	Social security	Health + social security
Main focus	Social and functional support	Clinical support, rehabilitation, and care
Type of users	Elderly, children, people with disabilities, families	Dependent individuals or those with chronic illnesses
Location	ERPI, SAD, day centers, etc.	Clinical units or home
Professionals	Social workers, assistants, etc.	Doctors, nurses, therapists, etc.
Duration of care	Generally permanent	Temporary or extended, but with specific criteria
Example of overlap	An elderly person in an ERPI may also receive care from the RNCCI (e.g., rehabilitation after a stroke)

**Table 2:** Long-Term Care (LTC): international comparison.

Criterion	Brazil	Portugal	Spain
Definition	Prolonged support for people with functional loss (elderly, people with disabilities)	Clinical and social support for people with functional dependency	Support for people in dependency situations (Dependency Law)
Main legal framework	No specific national law; SUS and SUAS norms are complementary	Law N°. 101/2009 (RNCCI) and Social Security legislation	Law 39/2006 – Law for the Promotion of Personal Autonomy and Care for Dependency
Public system involved	SUS (Health) + SUAS (Social Assistance)	SNS (Health) + Social Security	National Health System (SNS) + IMSERSO + Social services from Autonomous Communities
Care focus	Institutional care (nursing homes), home care, caregiver support	Rehabilitation, medical and ongoing social care	Continuous personal and social care (home, residential, teleassistance)
Typical services	ILPIs (Long-Term Care Institutions), SAD, PCDs, CAPS, multidisciplinary teams	ERPI (residences), Day Centers, Continued Care (RNCCI), SAD	Residences, Day Centers, Home Help, Personal Assistance
Home care	SAD + primary care + home care via SUS	RNCCI teams + SAD + support from IPSS	SAD (Home Help Service) + personal assistance
Institutional care	ILPIs (public, philanthropic, private)	ERPI (residences) + ULDM (prolonged clinical care)	Geriatric residences (public, contracted, private)
Funding	Mixed: public (SUS/SUAS), philanthropic and private (direct payment)	Mixed: State co-financing, user may have co-payment	Mixed: co-payment based on income, direct subsidies
Support to informal caregiver	Limited: policies under development, no national statute	Informal Caregiver Statute (still limited in application)	PECEF (subsidy), technical and legal assistance to the caregiver
Dependency assessment	Not standardized nationwide, varies by municipality	Assessment by RNCCI multidisciplinary team + family doctor	Official 3-level dependency assessment (dependency scale)
Decentralization	High: municipalities and states define policies and structures	Medium: SNS and Social Security with strong national presence	High: each Autonomous Community manages services locally
Main challenges	High demand, underfunding, lack of clear national regulation	Waiting lists, staff shortages, unequal coverage	Long waiting lists, regional inequalities, administrative complexity

In this context, the Portuguese government contributes with financial support and healthcare services through the National Health Service (SNS), contributing to the provision of integrated long-term care for the dependent older population, including prevention, rehabilitation, and palliative care actions.^19^, ^20^ However, the applicability and scope of these policies are still being debated in light of the longevity scenario and the growing demand for care in the country.^21^, ^22^ Since 2019, Portugal has had a Statute of the Informal Caregiver (ECI), created by Law No. 100/2019, of September 6, and regulated by Ordinance No. 64/2020, which provides for: official recognition, psychological support and training, as well as financial assistance (a monthly benefit of €560.19 starting January 1, 2025). It also establishes priority access to health and social support services, as well as work-life balance measures (such as flexible working conditions) for all individuals who serve as Informal Caregivers.^23^, ^24^

In Spain, long-term care (LTC) has traditionally been based on principles of mutual support among families and local communities, particularly religious orders. However, as in Portugal, specific public policies have been established for this area. In this context, the implementation of the Law for the Promotion of Personal Autonomy and Care for People in Situations of Dependency marked a significant shift, progressively transferring the responsibility of care to the State through the definition of rights, access criteria, and public funding of services.^4^, ^25^

The range of services in Spain is broad, including day centers and residential facilities that provide comprehensive health care and social activities for older adults. This offering may vary from one autonomous community to another, with the Basque Country, Navarra, and Catalonia being the regions that provide the most socioeconomic support. While day centers allow older adults to receive care during the day and return home at night—preserving family ties—residential facilities offer continuous care in a safe and assisted environment.^26^ Home care is another pillar of LTC policies in Spain and includes emergency services or social assistance through electronic devices, as well as support with daily activities such as feeding, personal hygiene, and mobility.^27^

Another advancement is that Spain recognizes the importance of informal caregivers, offering financial benefits based on the degree of dependency of the older person and the caregiver's socioeconomic situation. These benefits can reach a monthly amount of €1069.80 in Navarra, €911 in the Basque Country, or €715.07 in Catalonia. In addition, training programs and psychological support are available for these caregivers, with the aim of improving their skills and alleviating the emotional burden associated with caregiving.^28^, ^29^

However, despite major advancements, the long-term care (LTC) system in Spain faces significant challenges, such as financial sustainability and regional inequality in access to these services.^30^, ^31^

When comparing the three contexts, similar challenges can be observed: the growing number of older, long-lived, and frail individuals; financial sustainability; the availability of social policies; social prestige and recognition; and the technical and human training required for care provision.^32^

Nevertheless, it is observed that while Portugal and Spain have made progress in regulating and structuring specific public care policies – with legal recognition and service provision (Table 3) – Brazil still faces challenges in consolidating a coordinated and sustainable national policy.^33^

**Table 3:** Comparison between Portugal and Spain.

Characteristic	Spain	Portugal
Legal framework	Law 39/2006	Law N°. 101/2009 (RNCCI) + social responses from RSES
Management	Decentralized by Autonomous Communities	Shared management (Ministry of Health + Social Security)
Assessment	3 levels of dependency	Functional autonomy scales + RNCCI decision
Home care	SAD + personal assistance	SAD + continued care teams
Institutional care	Geriatric residences (public, contracted)	ERPI and ULDM (RNCCI)
Financial support for families	PECEF (informal caregiver)	Informal Caregiver Statute (less comprehensive)

According to Table 4, in the countries analyzed, Europe, particularly Portugal have already surpassed a life expectancy at birth of 80 years. However, a temporary decline was observed in 2020 and 2021, particularly associated with the COVID-19 pandemic, followed by partial recovery.^34^ In Brazil, according to projections from the Brazilian Institute of Geography and Statistics (IBGE),^35^ That threshold will be reached in 2050, at which point one in every four people will be an older adult. Two components stand out in this regard: Europe and Portugal likely benefited from post-World War II welfare state policies in the 20th century, which allowed for more prominent aging, while Brazil experienced a more accelerated demographic and epidemiological transition, which intensified after 1980 with declining birth rates and increased life expectancy. However, according to projections, this acceleration will continue, so that in just under 25 years, the proportion of older adults will rise from just over 15% in 2025 to slightly more than 25% in 2050. In all countries, higher life expectancy among women compared to men can be observed, highlighting the feminization of old age.

**Table 4:** Life expectancy and crude mortality indicators for Brazil, Spain, and Portugal, OECD.

Life expectancy and mortality	Year		
*Life expectancy at birth*	2019	2020	2021
Brazil	75.3	74	72.8
Portugal	81.9	81.3	81.6
Spain	84	81.1	81.8

*Difference in life expectancy at birth (women minus men)*	2019	2020	2021
Brazil	6.3	6.7	6.4
Portugal	6.1	6.1	5.9
Spain	5.6	5.6	5.8

*Preventable mortality per 100,000 inhabitants*^a^	2019	2020	2021
Brazil	188.8	260.2	357.2
Portugal	112.4		
Spain	92.4	118.4	111.6

*Treatable mortality through healthcare interventions per 100,000 inhabitants*^b^	2019	2020	2021
Brazil	140.5	133.7	138.5
Portugal	65		
Spain	52.2	51.4	50.6

*Total preventable and treatable mortality per 100,000 inhabitants*^c^	2019	2020	2021
Brazil	329.3	393.9	495.7
Portugal	177.4		
Spain	144.5	169.9	162.1

a Preventable mortality is defined as causes of death among people under the age of 75 that can be avoided primarily through effective public health interventions and primary prevention (that is, before the onset of disease or injury, to reduce incidence).^35^

b Treatable (or amenable) mortality is defined as causes of death that can be avoided primarily through timely and effective healthcare interventions, including secondary prevention and treatment (that is, after the onset of disease, to reduce lethality).^35^ ^c^ Sum of preventable and treatable mortality.^35^

Regarding the mortality profile, Brazil, even before the COVID-19 pandemic in 2019, already showed more than twice the number of preventable and treatable deaths compared to Spain—a figure that exceeded a threefold difference in 2022. Brazil's performance was also 1.8 times worse than that observed in Portugal in 2019. Spain presents the lowest mortality indicators among the three countries with universal health systems.

The worst statistics, both in terms of mortality and life expectancy, may be associated with the COVID-19 pandemic. Although it is not possible to analyze the case of Portugal due to the absence of data recorded by the OECD, Brazil showed a poorer response to the pandemic compared to Spain, which is reflected both in the decline in life expectancy and in the sharp increase in preventable deaths, mostly associated with primary care.

If the future is the sum of present efforts, in Brazil these efforts have been directed toward defending health as a right for all and a duty of the State. In this context, it is important to highlight the challenges related to the financial and economic sustainability of the Unified Health System (SUS), especially following Constitutional Amendment 96, which in 2016 proposed a freeze on health spending. Another possible factor influencing these statistics lies in the sociopolitical tensions surrounding the management of the COVID-19 pandemic, marked by delays in providing the population with scientifically proven vaccines and treatments.

In this context, it is observed that the Brazilian universal health system faces challenges related to the management, operationalization, and planning of healthcare actions. According to Paim (2018),^36^ it is the largest public health system in the world in terms of territory and population, covering more than 5000 municipalities and serving approximately 75% of Brazil's more than 210 million inhabitants.

In the ongoing struggle to defend a democratic SUS, following the government transition in 2022, a decree was enacted repealing Constitutional Amendment 96, thereby increasing fiscal support for SUS. However, there is still a long road ahead, especially in the face of population aging and the growing demand for long-term care. The outlook will certainly not be promising if decisions related to care are not made assertively and proactively.

From an epidemiological perspective, according to Table 5, Spain showed a higher prevalence of deaths from neoplasms and dementia syndromes, while Brazil recorded higher mortality rates from cerebrovascular diseases and COVID-19. Mortality rates from dementia in Spain and Portugal were approximately ten times higher than those observed in Brazil, while mortality rates from cerebrovascular diseases in Brazil were nearly double those recorded in Spain. Nevertheless, given the scenario of accelerated population aging, a significant increase in neoplasms and dementias is expected in Brazil, which will require social responses in the short, medium, and long term.

**Table 5:** Crude mortality rates by specific causes in Brazil, Spain, and Portugal, OECD.

Mortality rates	Year		
*Mortality rate from neoplasms per 100,000 inhabitants*	2019	2020	2021
Brazil	172.6	162.8	162.1
Portugal	210.4		
Spain	195.8	192.5	191.1

*Mortality rate from dementias per 100,000 inhabitants*	2019	2020	2021
Brazil	3.6	2.7	3.3
Portugal	33.3		
Spain	31.5	29.3	27.1

*Mortality rate from cerebrovascular diseases per 100,000 inhabitants*	2019	2020	2021
Brazil	82.5	77.6	77.8
Portugal	71.5		
Spain	39.7	39.5	37.5

*Mortality rate from COVID-19 per 100,000 inhabitants*^a^	2019	2020	2021
Brazil		158.1	282.6
Portugal			
Spain		115.2	62.9

a Mortality rates are crude rates per 100,000 inhabitants and are not age-standardized.

It should be noted that the mortality rates presented are crude and not age-standardized, which may partly explain the higher mortality from dementias observed in countries with older population structures, such as Spain and Portugal. Nevertheless, given the scenario of accelerated population aging, a substantial increase in neoplasms and dementias is expected in Brazil, which will require social responses in the short, medium, and long term.

Regarding the organization of service delivery systems, OECD statistics^34^ indicate that the formal employment rate in long-term care in Spain was 4.8 per 100 inhabitants aged 65 years and over in 2019, and 5.4 in 2022. In Portugal, it was 0.8 in both 2019 and 2022. In Brazil, although there are no consistent data on the proportion of professionals working in long-term care relative to the number of older adults, there are approximately 3167 geriatricians nationwide, which corresponds to 1.49 professionals per 100,000 inhabitants. This professional group represents 0.7% of medical specialists in Brazil.^37^

Regarding professionals specialized in Gerontology, in 2023 there were just over 487 certified specialists by SBGG^38^ and 11 stricto sensu graduate programs dedicated to training Master's and Doctoral students in Gerontology.^39^ In Brazil, only three universities offer undergraduate degrees in Gerontology. Taken together, these data reinforce the need to invest in the training and qualification of professionals capable of working in the field of aging.

Other data, related to the number of LTC institutions, according to OECD data from 2022, indicate that in Spain there were: 1406 public institutions (25.18%), 1975 philanthropic institutions (35.37%), and 2202 private for-profit institutions (39.40%). In Portugal, the distribution was: 7 public institutions (1.8%), 310 philanthropic institutions (80.51%), and 78 private for-profit institutions (20.25%).^40^

In Brazil, according to a survey conducted by Domingues et al. (2021),^41^ there were 2381 long-term care institutions (ILPI) registered in the Unified Social Assistance System (SUAS). After consolidating and filtering data from multiple sources, a total of 7029 institutions were identified nationwide. According to the data presented by the authors, the total number of institutions represents a 105% increase compared to the census conducted by Camarano (2010),^42^ of which 65% were philanthropic institutions.^41^, ^42^

In the most recent survey conducted by Domingues et al. (2021)^41^ it was not possible to estimate the percentage of Brazilian institutions that are public, private, or philanthropic. However, assuming that the percentage of philanthropic institutions has remained stable, it is observed that this percentage was also relatively high in Portugal and lower in Spain (approximately one-third). Spain stands out for the greater presence of public institutions, which indicates State support in the provision of care. Nonetheless, it remains to be determined how these services are offered and whether there is any level of user co-participation to enable the operation of public institutions.^41^

## Conclusion

The analysis of long-term care (LTC) for older adults in the three countries indicated that Brazil needs to make progress in developing policies directed at older individuals with functional limitations. Spain shows better indicators of care organization compared to Portugal, which may be related to how long-term care policies are structured, including the provision of home care services, day centers, and financial support for caregivers. However, in the face of population aging, Spain also faces challenges in maintaining the sustainability of care. It is important to note that a comparative analysis between the three countries is approximate, as there are socioeconomic, political, and sociocultural differences that must be taken into account. Nevertheless, the findings suggest an urgent need for improved organization of LTC in Brazil.

**What is known about the topic?**

• Population aging increases the demand for long-term care (LTC), posing challenges to universal health systems.
• LTC models vary: in less structured contexts, family care predominates; in more advanced settings, formal care networks (home care, day centers, institutions) are developed.
• Spain and Portugal have developed legal frameworks and services for dependency; Brazil remains fragmented, centered on family care and long-stay institutions (ILPI).

**What does this study contribute?**

• Compares LTC policies across three universal systems (Brazil, Spain, and Portugal), linking legal frameworks, service provision, and OECD indicators.
• Highlights that Spain's better organization (home care, day centers, support for caregivers) contrasts with Portuguese limitations and Brazilian gaps.
• Demonstrates the urgency for Brazil to structure a national LTC policy for people with functional limitations, reorganizing service provision and investing in human resource training.

Own authorship (2025).

## Authorship statement

We further declare that all authors fully met the authorship criteria established by the journal, contributing significantly to the scientific and intellectual development of the work, approving the final version of the manuscript, and assuming public responsibility for its content.

## Ethical considerations

This is a documentary and bibliographic study based on public data and is therefore exempt from submission to a Research Ethics Committee, in accordance with Resolution No. 510/2016 of the National Health Council (CNS).

## Declaration of generative AI and AI-assisted technologies in the manuscript preparation process

We confirm that no Artificial Intelligence (AI)-based tools were used at any stage of the manuscript's preparation. All activities related to the conception, writing, analysis, and revision of the text were carried out exclusively by the authors, manually and independently.

## Funding

This work was supported by the Foundation for Research Support of the Federal District (FAPDF) [Process Number: 00193-00002043/2023-78].

## Conflict of interest

Letycia Parreira de Oliveira declarer ports financial support was provided by Foundation for Research Support of the Federal District. Reports a relationship with that includes: funding grants. Has patent pending to. If there are other authors, they declare that they have no known competing financial interests or personal relationships that could have appeared to influence the work reported in this paper.
